# Visuospatial cueing differences as a function of autistic traits

**DOI:** 10.3758/s13414-024-02871-0

**Published:** 2024-04-01

**Authors:** Min Quan Heo, Michael C. W. English, Murray T. Maybery, Troy A. W. Visser

**Affiliations:** School of Psychological Science, University of Western Australia, 35 Stirling Highway, Crawley, WA 6009 Australia

**Keywords:** Attention, Cognitive neuroscience, Cognitive and attentional control

## Abstract

Atypical orienting of visuospatial attention in autistic individuals or individuals with a high level of autistic-like traits (ALTs) has been well documented and viewed as a core feature underlying the development of autism. However, there has been limited testing of three alternative theoretical positions advanced to explain atypical orienting – difficulty in disengagement, cue indifference, and delay in orienting. Moreover, research commonly has not separated facilitation (reaction time difference between neutral and valid cues) and cost effects (reaction time difference between invalid and neutral cues) in orienting tasks. We addressed these limitations in two experiments that compared groups selected for Low- and High-ALT levels on exogenous and endogenous versions of the Posner cueing paradigm. Experiment 1 showed that High-ALT participants exhibited a significantly reduced cost effect compared to Low-ALT participants in the endogenous cueing task, although the overall orienting effect remained small. In Experiment 2, we increased task difficulty of the endogenous task to augment cueing effects. Results were comparable to Experiment 1 regarding the finding of a reduced cost effect for High-ALT participants on the endogenous cueing task and additionally demonstrated a reduced facilitation effect in High-ALT participants on the same task. No ALT group differences were observed on an exogenous cueing task included in Experiment 2. These findings suggest atypical orienting in High-ALT individuals may be attributable to general cue indifference, which implicates differences in top-down attentional processes between Low- and High-ALT individuals. We discuss how indifference to endogenous cues may contribute to social cognitive differences in autism.

**Supplementary Information:**

The online version contains supplementary material available at 10.3758/s13414-024-02871-0.

## Introduction

Orienting refers to the act of involuntarily (exogenously) or voluntarily (endogenously) directing one’s attention to objects or locations in space (Posner & Petersen, 1990). It is a skill that develops early in life, with infants displaying goal-directed orienting as early as 4 months of age (Hendry et al., 2019; Johnson et al., 1991), and is a pivotal milestone because it affects the selection of sensory elements in the environment that shape cognitive processing (Lellis et al., 2013).

Studies of endogenous orienting typically use the spatial cueing task pioneered by Posner (1980) in which attention orienting is prompted by the appearance of a centrally presented directional cue (e.g., an arrow) that signals the potential location of an upcoming target. This directional cue can be valid (pointing in the direction where the target appears) or invalid (pointing in a different direction to where the target appears). By comparison, spatial cueing tasks examining exogenous orienting use brief and sudden cues (e.g., a flash or brightening of peripheral space) that appear at the location of an upcoming target (valid) or at a different location (invalid) and do not contain any directional meaning requiring interpretation.

Conventionally, participants in both endogenous and exogenous cueing tasks respond more quickly and accurately to targets presented on validly cued trials than on invalidly cued trials. This has been attributed to the fact that valid cues correctly allow participants to orient attention to the location of the upcoming target, benefitting its perception, while invalid cues draw attention to an unhelpful location, requiring time-consuming disengagement and then re-engagement of attention at the location of the target after it appears (Muller & Rabbitt, 1989). Notably, however, the underlying mechanisms are different, reflecting a slower volitional process in the case of endogenous cues and a faster non-volitional process in the case of exogenous cues (Chica et al., 2013).

## Orienting of attention in autism

Atypical visual orienting has been linked to a number of neurodevelopmental conditions including autism, a condition primarily characterised by generalised, persistent difficulties in social interaction and communication, and the presence of restricted, repetitive patterns of behaviours, interests and activities (American Psychiatric Association, 2013). Many studies have shown atypical orienting in autistic children (Helminen et al., 2017; Riddiford et al., 2022), which Keehn et al. (2013) suggest may be a precursor for difficulties in joint attention between caregivers and children, and potentially broader social development problems later in life. Atypical orienting is also observed in individuals with a high level of autistic-like traits (ALTs), though without the presence of functional difficulties (Elsabbagh et al., 2013). This suggests that atypical orienting may be a perceptual characteristic in which high-ALT individuals are qualitatively similar to individuals with autism (Baron-Cohen et al., 2001; Constantino & Todd, 2003).

Competing theories have been put forth to account for orienting behaviour observed in individuals with autism or high ALT. In one theory, atypical orienting is thought to arise from difficulties disengaging attention from invalidly cued locations (Zwaigenbaum et al., 2005). For example, Landry and Bryson (2004) compared orienting performance using an eye-tracker for infant siblings of autistic children (‘high-risk’ infants) and typically developing children (‘low-risk’ infants). On each trial, infants were first presented with an engaging central stimulus followed by an equally engaging peripheral stimulus. The results indicated that the ‘high-risk’ infants took longer to move their eyes from the central to the peripheral stimulus than did the ‘low-risk’ infants. Similar results were reported by Elsabbagh et al. (2013), who found that longer disengagement latencies at 14 months were associated with a higher risk of being diagnosed with autism, and that infants who eventually went on to be diagnosed with autism at the age of 3 years showed no improvement in their ability to disengage. However, evidence of poor disengagement remains limited to studies that sampled very young children. Moreover, several studies have failed to find any endogenous orienting differences between autistic and non-autistic adults (Fan et al., 2012; Grubb et al., 2013). It is unclear whether this is due to age-related changes in orienting behaviour or to modifications made to the standard Posner paradigm used by studies that sampled young children in order to make the task more amenable to them. As a result, the conditions under which poor disengagement presents (that is, within a certain age group or under particular task conditions) remain unclear, given that these modifications to the standard Posner paradigm have made it difficult to distinguish between endogenous and exogenous cueing.

Another theoretical explanation for atypical endogenous orienting in autistic individuals is cue indifference. Adapting the paradigm used by Landry and Bryson (2004), Elsabbagh et al. (2009) added trials in which the central stimulus disappeared before the peripheral stimulus was presented, with the disappearance acting to ‘cue’ infant participants to anticipate and prepare their saccade for the subsequent stimulus. Elsabbagh et al. (2009) found that ‘low-risk’ infants exhibited shorter latencies when the central stimulus disappeared as compared to when the central stimulus remained on the screen, while ‘high-risk’ infants exhibited similar latencies whether or not the central stimulus disappeared. This suggests ‘high-risk infants’ were less able to process the cueing information provided by disappearance of the central stimulus. Similarly, Wainwright-Sharp and Bryson (1993) reported that individuals with or at risk of developing autism had slower reaction times (RTs) and reduced accuracy on valid trials in an endogenous cueing task compared to neurotypical individuals, suggesting that they were less influenced by cues even when potentially beneficial to task performance. Such findings, however, were not consistently replicated across studies examining cue indifference (Iarocci & Burack, 2004). Moreover, it is unclear whether a similar indifference would occur for exogenous cues.

A third theoretical explanation, based on studies examining exogenous orienting, was proposed by Townsend et al. (1996), who hypothesised that autistic individuals do not have impaired visual orienting per se, but rather orient more slowly than their non-autistic peers. This account was based on numerous experiments demonstrating poorer orienting performance in autistic individuals at a shorter cue-target stimulus onset asynchrony (SOA) that began to normalize as SOA lengthened (Harris et al., 1999; Townsend et al., 1996). However, in later work, Haist et al. (2005) reported that autistic individuals exhibited reduced activation of neural regions and different patterns of activation across neural regions for both short and long cue-target SOAs compared to their neurotypical counterparts, suggesting that they may be employing qualitatively different processing styles across varying SOAs, rather than merely responding more slowly.

## Limitations of existing evidence

**Fig. 1:**
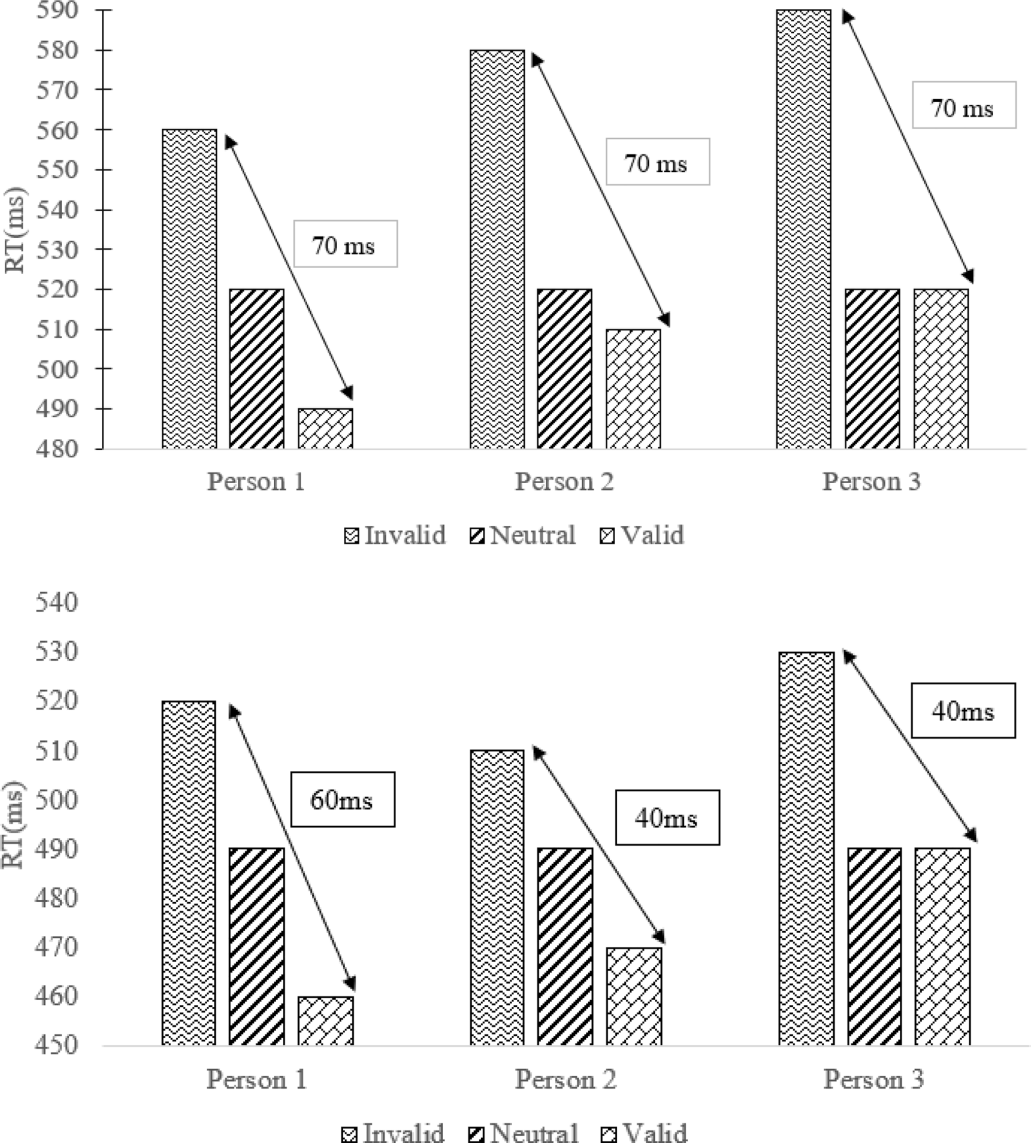
Illustration of how the conventional subtraction method is unable to separate different patterns of visuospatial orienting (**top panel**) and distinguish between theoretical models (**bottom panel**). In the top panel, the subtraction method yields the same orienting effect for Persons 1–3 despite clear differences in corresponding facilitation and cost effects. In the bottom panel, despite the same orienting effect for Persons 2 and 3, they are showing different patterns of cost and facilitation effects that are consistent with different theories, relative to Person 1

As the discussion above suggests, the literature on atypical orienting in autism is rife with competing theories and inconclusive evidence, and it is also unclear whether existing theoretical accounts could explain orienting behaviour broadly or apply only to either endogenous or exogenous cueing. In the present work, we examine whether some of this inconsistency may stem from methodological properties of the cueing experiments typically employed in this literature. Importantly, past studies have almost exclusively looked at cueing costs and benefits by subtracting target RTs on valid trials from those on invalid trials. While a common approach, the lack of a neutral cue condition – trials on which a cue is presented but does not provide target location information – means that no measure of baseline performance is obtained, and thus cue-related costs (slower responses arising from the presentation of an invalid cue) and facilitation (faster responses arising from the presentation of a valid cue) are seldom isolated. The consequences of this omission are illustrated in the top panel of Fig. 1, which shows the performance of three hypothetical individuals presenting with different cost (Person 1 = 40 ms, Person 2 = 60 ms, Person 3 = 80 ms) and facilitation effects (Person 1 = 30 ms, Person 2 = 10 ms, Person 3 = 0 ms). While each of these hypothetical individuals is clearly responding to cues differently, when the conventional method of subtracting RTs on valid trials from RTs on invalid trials is used, all three show an identical orienting effect of 70 ms.

In the context of testing theoretical explanations for atypical orienting, the importance of separately assessing cue-related cost and facilitation becomes clear. For instance, Wainwright-Sharp and Bryson’s (1993) findings of slower RTs and reduced accuracy on valid trials in autistic individuals relative to non-autistic individuals appears, at first glance, to be consistent with the theory of general cue indifference. However, as illustrated by the hypothetical data shown in the bottom panel of Fig. 1, this may not necessarily be the case. Here, Persons 2 and 3 show the same reduced orienting effect (calculated using the conventional subtraction method) compared to Person 1, but Person 2’s performance is consistent with general cue indifference (reduced cost and reduced facilitation compared to Person 1), while Person 3’s performance is consistent with disengagement difficulties (greater cost compared to Person 1).

A study by Keehn at al. (2010) has partially alleviated this issue by enabling separation of cost and facilitation through the introduction of a null-cue condition in which trials contained no cues. However, the null-cue condition is critically different from a neutral condition because the omission of the cue also eliminates alerting information about the upcoming presentation of the target that is maintained in the neutral condition by presenting a spatially uninformative cue (Hamame et al., 2008). Consequently, comparing performance on null cue trials with performance on valid or invalid cue trials would likely reflect influences of both spatial cueing and alerting, and would not permit their separation.

Another obstacle to evaluating theoretical accounts for atypical orienting is that they are largely based on outcomes from studies of atypical orienting patterns in young, autistic (or high-risk) children. Studies conducted on the adult population have mostly failed to report any orienting differences between autistic and non-autistic individuals (Fan et al., 2012; Grubb et al, 2013). This picture is further complicated because of the different cueing paradigms used across studies. Fan et al. (2012), for instance, used the Attention Network Test, which is more complex and demanding in nature than the Posner tasks described above. With this in mind, our work focuses exclusively on examining atypical orienting in adults using variations of the Posner task.

Finally, some discrepancy in the literature could also be due to the inconsistent cue-target SOAs adopted across studies. For example, some studies using SOAs of 100 ms and 800 ms for comparison (e.g., Landry & Parker, 2013) reported results not consistent with the delayed orienting hypothesis. However, a study by Flanagan et al. (2015) using SOAs of 170 ms and 650 ms found that autistic individuals exhibited orienting difficulties only at the shorter SOA, consistent with the delayed orienting hypothesis. This comparison highlights the potential importance of SOAs, as they may influence the nature of the orienting differences observed between autistic and non-autistic individuals. To address this issue, we use multiple and consistent cue-target SOAs across our experiments.

In summary, existing studies vary significantly in terms of participant age, and in the cueing conditions and SOAs used. This heterogeneity makes it particularly challenging to empirically distinguish between existing theories, which is compounded by the fact that most studies do not separately assess for cue-related cost and facilitation, making it difficult to isolate the specific processes underlying any orienting differences observed. Existing studies typically subtracted RT on valid trials from RT on invalid trials, and used the resulting difference as an indicator of orienting performance, with a smaller difference suggesting poorer orienting ability (Allen & Courchesne, 2001). However, current theories argue that orienting difficulties may occur because of poor performance on invalid trials (cost effect) but not valid trials (due to difficulty in disengaging attention from the incorrectly cued location), or instead that other patterns of atypical cost and facilitation could be observed. These predictions can only be tested effectively by comparing validly and invalidly cued trials to a neutral condition that presents a spatially uninformative cue.

## The present study

In our first study we investigated the nature of endogenous orienting differences between individuals with Low and High ALTs. To better distinguish between existing theoretical accounts, we included valid, invalid and neutral trials such that facilitation and cost effects could be independently examined. To address the delayed orienting account and whether orienting differences in those with High ALTs start to normalise at some point after the cue is presented, the current study also included three SOAs (200 ms, 500 ms and 800 ms) to establish a time course of visual orienting using the measures of facilitation and cost effects.

With respect to outcomes, the disengagement account predicts increased cost effects but similar facilitation effects for High-ALT individuals compared to Low-ALT individuals due to specific difficulties disengaging attention from cued locations on invalidly cued trials. According to the cue indifference account, reduced cost and facilitation effects would be expected in High-ALT individuals for whom endogenous cues do not lead to the same level of orienting as for their Low-ALT peers. Finally, the delayed orienting account predicts differences in either the cost or facilitation effects between High- and Low-ALT individuals at shorter SOAs that decrease as the cue-target SOA increases.

## Experiment 1

### Method

#### Participants

The two experiments in this study were carried out in accordance with procedures approved by the Human Research Ethics Office at the University of Western Australia (UWA). Potential participants for each experiment were briefed about the nature and purpose of the research and informed consent was collected prior to commencing the study. A total of 197 UWA psychology undergraduates (133 female, 64 male), ranging in age from 18 to 35 years (*M* = 20.3, *SD* = 3.8) completed the questionnaire to screen for ALT level.

#### Materials 

ALT level was assessed using the 50-item self-report Autism Spectrum Quotient questionnaire (AQ; Baron-Cohen et al., 2001). Each item on the AQ has four responses with ascribed scores of 1–4 according to the scoring procedure introduced by Austin (2005), with a higher score indicating more pronounced autistic traits. This scoring method was selected based on Stevenson and Hart’s (2017) findings that the 1–4 scoring procedure was superior to the binary scoring method originally proposed by Baron-Cohen et al. (2001) in relation to internal consistency and reliability. The use of a scoring system matching the Likert rating scale, such as Austin’s (2005) system, was also strongly recommended for assessing the level of autistic traits in neurotypical adults due to its ability to retain detail in responses and increase variability in scores (Stevenson & Hart, 2017). The AQ has acceptable internal consistency, with a Cronbach alpha coefficient of .75 (Broadbent et al., 2013). Strong test-retest reliability was also shown, with a .77 correlation between AQs completed 2 weeks apart (Baron-Cohen et al., 2001).

The endogenous cueing task was administered using Presentation software (Neurobehavioral Systems) on a Windows computer attached to a 20.7-in. (w) × 11.7-in. (h) monitor (Dell P2419H) running at a refresh rate of 60 Hz. Participants were seated approximately 50 cm from the monitors. The background colour of the screen was set to RGB 255, 255, 255 (white). A fixation cross measuring 0.1° × 0.1°, as well as two placeholder squares measuring 4.5° × 4.5°, were used. The fixation cross was positioned on the centre of the screen, while the placeholder squares were positioned on the left and right sides of the screen and spaced 8° apart from the fixation cross. Left-pointing, right-pointing and double-ended arrows served as cues and measured 2.3° in length × 0.6° in breadth. The target was a triangular symbol measuring 0.8° × 0.8°, which could be oriented either upwards or downwards. The colour of the fixation cross, placeholder squares and cues were set to RGB 0, 0, 0 (black).

#### Procedure

The endogenous orienting task contained three cue conditions (invalid, neutral and valid) and three cue-target SOAs (200 ms, 500 ms and 800 ms). The three cue-target SOAs were determined from a pilot study conducted to investigate the magnitude of facilitation and cost effects at eight different SOAs. The pilot study showed that endogenous facilitation and cost effects were greatest at 200 ms, before declining after 500 ms, and were smaller but still evident at 800 ms. There were 20 trials at each SOA for each of the neutral and invalid cue conditions and 80 trials at each SOA for the valid condition, yielding a total of 360 trials. This ratio of valid to invalid cues is consistent with the majority of studies in the autism literature (e.g., Pruett et al., 2011; Senju et al., 2004). The experiment was divided into four equal-sized blocks, with opportunities for breaks provided at the end of each block. Before commencing the task, participants were instructed to report the orientation of the target (up, down) as quickly and accurately as possible, and that the cues would accurately predict the target location on 80% of trials.

**Fig. 2:**
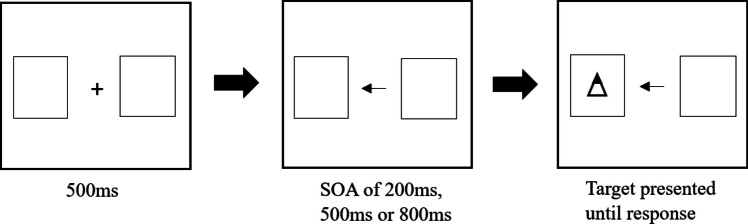
Sequence of events within a single trial in the endogenous cueing task

The sequence of a typical task trial is illustrated in Fig. 2. Each trial began with a 500-ms presentation of a fixation cross flanked by an empty ‘placeholder’ square on each side of the screen that designated the potential target locations. The fixation cross was replaced by a left-pointing, right-pointing or double-ended arrow presented for 200 ms, 500 ms or 800 ms. Finally, the target symbol, which consisted of a triangle pointing up or down, was presented inside one of the two placeholder squares, and remained on the screen until the participant indicated the orientation of the target using the ‘up’ or ‘down’ key. Once the participant had responded, the target disappeared while the cue was replaced by the fixation cross. This was followed by an inter-trial interval of 500 ms before the next trial was presented.

#### Design and analysis

Participants were selected for the Low- and High-ALT groups on the basis of their overall AQ scores from the screening (Low-ALT group = bottom 20% of scores; High-ALT group = top 20% of scores). This yielded a total of 77 participants, with 41 participants in the Low-ALT group and 36 participants in the High-ALT group.

Data from participants whose target discrimination accuracy, cost score or facilitation score was more than 2.5 SDs below their respective overall group means were excluded from analyses. This resulted in the exclusion of 23 participants (13 Low-ALT group, 10 High-ALT group) from further analyses.^1^ Descriptive statistics for the remaining participants can be seen in Table 1.

**Table 1:** Descriptive statistics for Low- and High-ALT groups for Experiment 1

	Low ALT		High ALT	
*N*	28 (19 female; nine male)		26 (14 female; 12 male)^a^	
	Age, y	ALT score	Age, y	ALT score
Mean	20.3	89.0	21.2	130
*SD*	4.1	5.3	3.4	9.7
Range	18–35	77–95	18–34	123–168

^a^While the ALT groups were not well matched for sex, key results remained unchanged when sex was included as an extra factor in the ANOVA on RTs

For the remaining participants, mean target accuracy was calculated separately as a function of ALT group (High vs. Low), SOA (200, 500, 800 ms) and Cue Condition (invalid, neutral, valid). In the analysis of mean RT, individual RTs (on correct trials) that were more than 2.5 SDs above or below each participant’s overall mean for each combination of cue condition and SOA were excluded from further analyses. This accounted for 6.65% of the total number of trials for the Low-ALT group and 6.92% of the total number of trials for the High-ALT group. Mean RTs were then calculated and analysed in the same manner as accuracy scores. IBM SPSS Statistics 26.0.0 was used to perform all statistical analyses.

### Results

Mean accuracy ranged from 95% to 98%. An ALT group × SOA × Cue Condition mixed-design analysis of variance (ANOVA) revealed no significant main effects, all *F* < 1.60, all *p* > .20, all *η*^*2*^ < .03, or interactions, all *F* < .41, all *p* > .75, all *η*^*2*^ < .002, suggesting interpretation of the RT data below is not compromised by speed-accuracy tradeoffs (see Appendix 1 for a detailed presentation of this analysis).

**Fig. 3:**
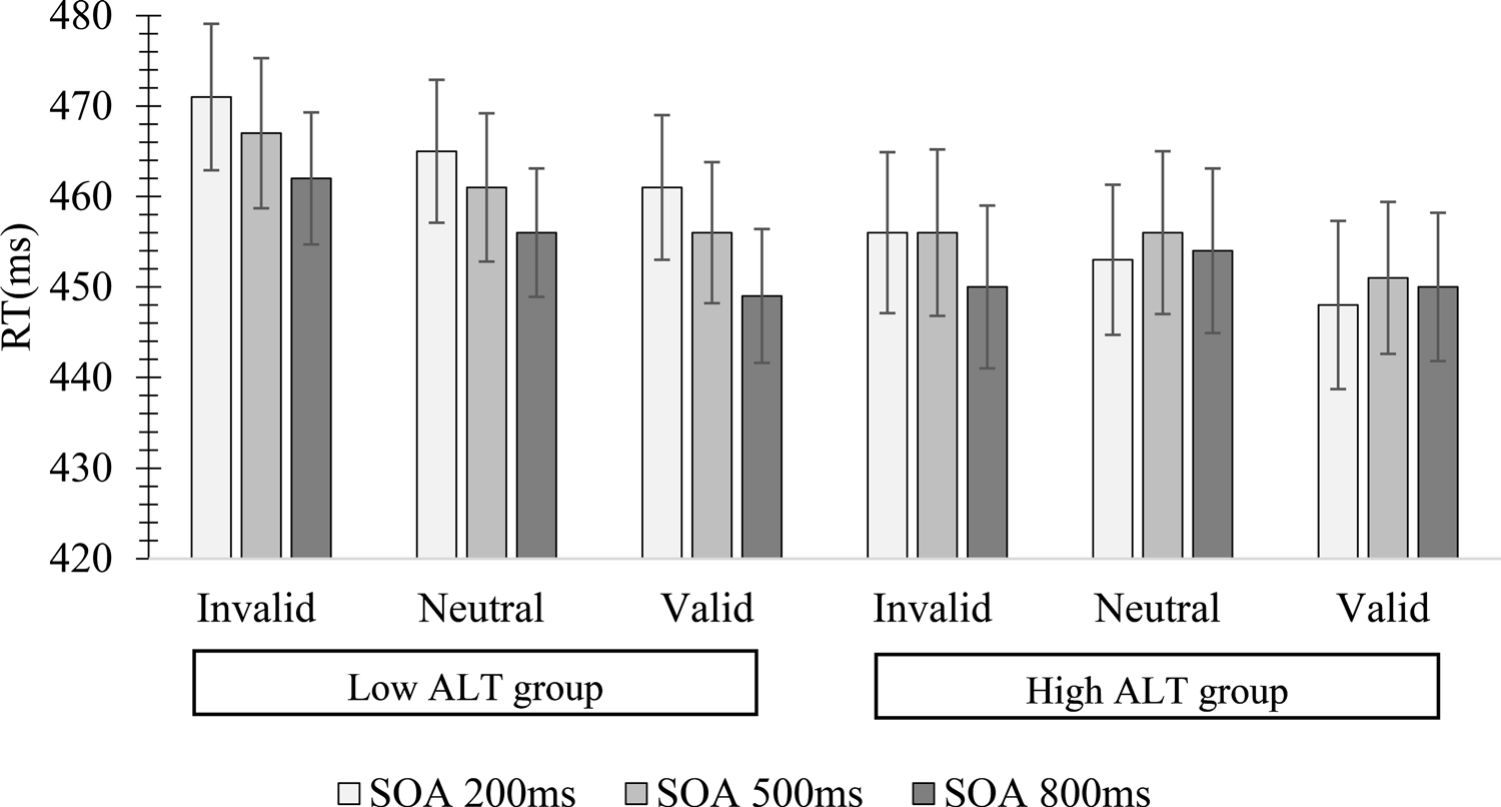
Mean reaction time (RT; ms) in Experiment 1 as a function of autistic-like trait (ALT) group, stimulus onset asynchrony (SOA), and Cue Condition. Error bars represent standard errors of the mean

An ALT group × SOA × Cue Condition ANOVA performed on mean RT yielded significant main effects of Cue Condition, *F*(2,104) = 23.81, *p* < .01, *η*^*2*^ = .25, and ALT Group, *F*(1,52) = 2.40, *p* = .03, *η*^*2*^ = .06. There was also a significant interaction between Cue Condition and ALT group, *F*(2,104) = 3.76, *p* = .04, *η*^*2*^ = .06. Post hoc t-tests revealed that RTs for the Low-ALT group were significantly slower than RTs for the High-ALT group on invalid trials, *t*(52) = 4.16, *p* = .02, *d* = .45, and neutral trials, *t*(52) = 3.27, *p* = .03, *d* = .28. No other main effects or interactions were significant, all *F*s < 1.10, all *p*s > .34, all *η*^*2*^s < .03 (see Fig. 3).

We next examined orienting using the conventional approach of subtracting mean RTs on valid trials from those on invalid trials separately for each combination of ALT group and cue-target SOA. These orienting scores were then subjected to an ALT group × SOA mixed-design ANOVA. This analysis revealed no significant main effects of SOA, *F*(2,104) = .38, *p* = .29, *η*^*2*^ = .002, or ALT Group, *F*(1,52) = .44, *p* = .60, *η*^*2*^ = .001, and no significant interaction, *F*(2,104) = .29, *p* = .87, *η*^*2*^ = .001. Thus, the conventional analysis suggested there were no differences in cueing across ALT groups or SOAs.

**Fig. 4:**
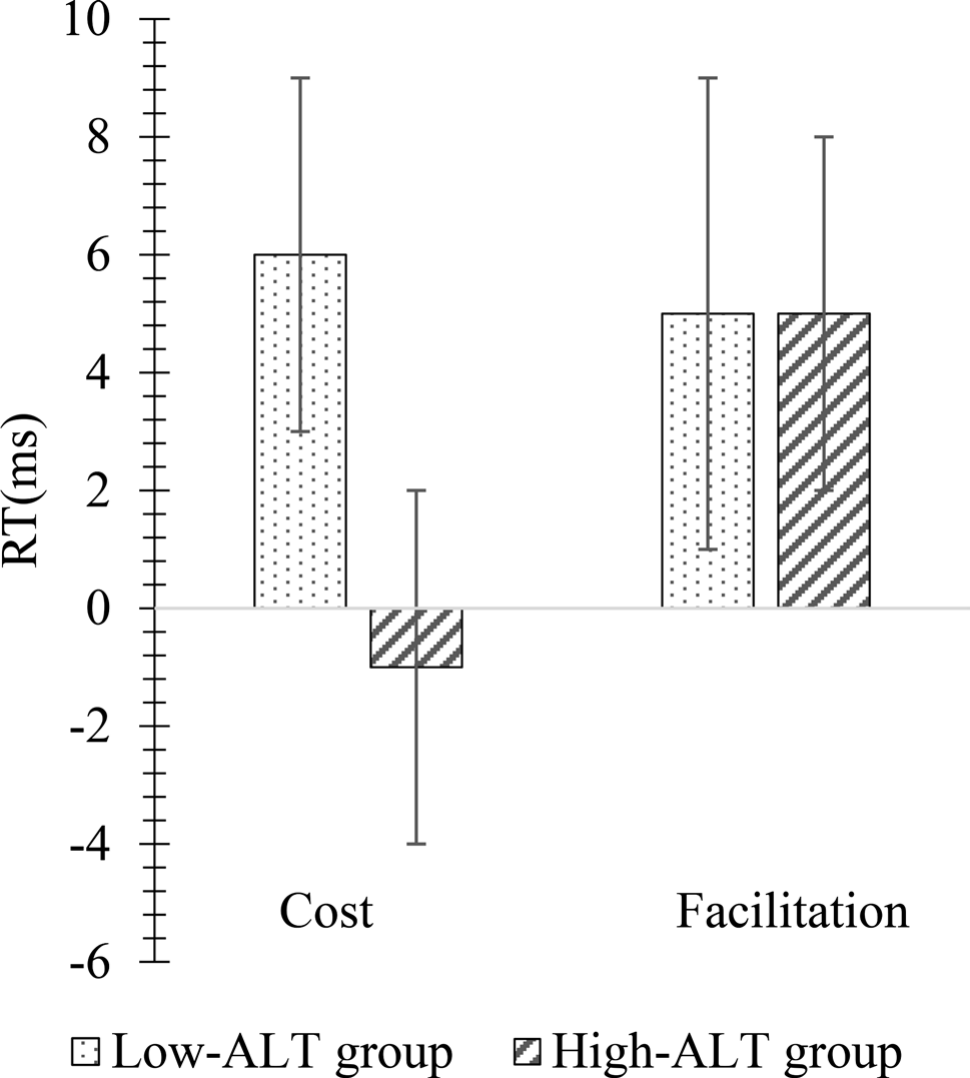
Cost and facilitation differences between High- and Low-autistic-like trait (ALT) groups in Experiment 1 (collapsed across stimulus onset asynchrony (SOA)). Error bars represent one standard error of the mean

Finally, we calculated mean facilitation and cost effects separately for each combination of ALT group and cue-target SOA. A facilitation effect was calculated by taking the difference between RTs on valid and neutral trials, while a cost effect was calculated by taking the difference between RTs on invalid and neutral trials. The resulting mean facilitation and cost effects, which can be seen in Fig. 4, were then submitted to an ALT group × SOA × Cue Influence (facilitation, cost) mixed-design ANOVAs.

There were no significant main effects of SOA or Cue Influence, all *F*s < 1.17, all *p*s > .59, all *η*^*2*^s < .03 (see Fig. 4). There was a significant main effect of ALT group, *F*(1,64) = 5.23, *p* = .04, *η*^*2*^ = .09, and a significant interaction effect of ALT group × Cue Influence, *F*(2,128) = 4.46, *p* = .04, *η*^*2*^ = .08. Post hoc t-tests showed that the facilitation effect was similar between both Low- and High-ALT groups, *t*(52) = 1.10, *p* = .49, *d* = .13, but the cost effect was significantly smaller for the High-ALT group, *t*(52) = 4.38, *p* = .03, *d* = .50. All other interactions were non-significant, all *F*s < .96, all *p*s > .76, all *η*^*2*^s < .01, were non-significant.

### Discussion

Experiment 1 examined whether individuals with varying levels of ALT showed different patterns of cost or facilitation on a Posner-type endogenous cueing task. The study aimed to examine evidence for three theoretical accounts of orienting differences associated with autism – difficulty with disengagement (Zwaigenbaum et al., 2005), overall cue indifference (Wainwright-Sharp & Bryson, 1993), and delayed orienting (Harris et al., 1999). Our key finding was a reduced cost effect in the High-ALT group compared to the Low-ALT group, such that the former group actually responded marginally faster on invalid trials (which required disengagement) relative to neutral trials.

With respect to the theoretical accounts noted earlier, this outcome is inconsistent with the pattern of findings (an increased cost effect for the High-ALT group) expected under the difficulty with disengagement account. However, it provides partial support for the cue indifference account, which suggests that individuals with High-ALT should be less impacted by invalid cues compared to those with Low ALT. That said, however, the cue indifference account also predicts a reduced facilitation effect should be found for the High-ALT group, which was not observed in the present experiment. Finally, the absence of any differential impact of SOA on cost or facilitation for the High-ALT group compared to the Low-ALT group is inconsistent with the delayed orienting account.

As we speculated in the Introduction, the conventional approach of measuring orienting performance in terms of the difference between valid and invalid trials yielded different results to those obtained from analysing facilitation and cost effects. The conventional analysis yielded no differences between Low- and High-ALT groups, despite the presence of a group difference in the cost effect. This highlights the importance of the inclusion of a neutral cue condition, which allowed us to index cue-related cost and facilitation effects more effectively, thereby allowing detection of group differences specifically in the cost effect.

Three other aspects of our data are also worth highlighting. First, the magnitude of the orienting effect in the present study appears to be much smaller than the estimate reported in a meta-analysis by Landry and Parker (2013). With a smaller orienting effect, it is correspondingly more difficult to observe possible influences of ALT group and SOA on cost and facilitation effects as well. Second, mean RTs are faster and mean accuracy is higher in the present study than in Landry and Parker’s (2013) meta-analysis. This raises the question of whether the endogenous task was perhaps too easy, which limited participants’ use of cue information, and perhaps limited the scope and generalizability of our findings. Lastly, High-ALT participants responded faster to targets than their Low-ALT peers. It is unclear whether this reflects more efficient processing, as has been found in other perceptual tasks (e.g., Ashwin et al., 2017; O'Riordan et al., 2001), or is the result of more limited use of cue information in the High-ALT group.

To address the possible ramifications of there being a ceiling effect Experiment 1, in our next experiment, we replicated the endogenous task of Experiment 1 except for using less-discriminable target stimuli with characteristics more similar to those reported in earlier studies (e.g., Flanagan et al., 2015; Wainwright-Sharp & Bryson, 1993). This change is also important to ensure our findings are generalisable. Additionally, we included an exogenous cueing task in Experiment 2 to allow us to directly compare endogenous and exogenous cost and facilitation effects in the same sample. Previous studies have yet to directly compare both endogenous and exogenous orienting within the same adult sample, or have done so in children with conflicting outcomes (Iarocci & Burack, 2004; Renner et al., 2006). Further, studies examining exogenous orienting have not isolated cost and facilitation effects, and it is possible that autistic or High-ALT individuals are atypical in either of these effects for exogenous and/or endogenous orienting.

## Experiment 2

**Fig. 5:**
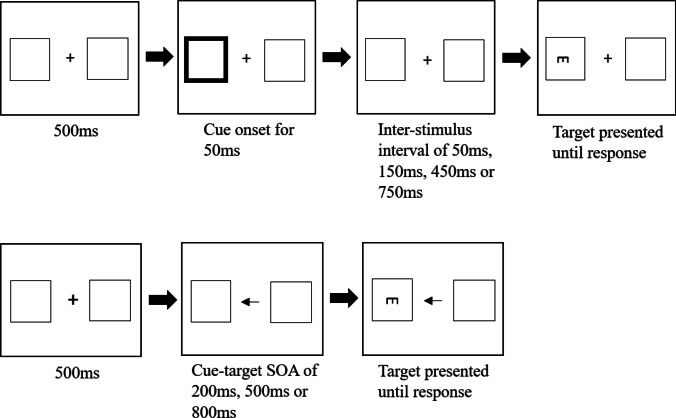
Sequence of events within a single trial for each task in Experiment 2. The top panel depicts the exogenous cueing task, while the bottom panel depicts the endogenous cueing task

The aims of Experiment 2 were twofold. The first was to increase the difficulty of the cueing task in order to potentially increase the magnitude of cost and facilitation effects. To this end, the target symbol was replaced with a smaller letter ‘E’ oriented in an upward or downward manner (see Fig. 5) to makes its visual properties more consistent with the target symbols used in previous studies. The second aim was to compare endogenous and exogenous cueing effects directly in the same participants. Thus, in addition to the endogenous orienting task, participants completed an exogenous orienting task in which the central arrow cue was replaced with a brief peripheral cue consisting of the brightening of one (or both) of the placeholder boxes that demarcated a potential target location.

### Method

#### Participants

A total of 220 UWA psychology undergraduates (149 female, 71 male), ranging in age from 18 to 35 years (*M* = 21.8, *SD* = 3.6) completed the AQ to screen for ALT level, as for Experiment 1.

#### Materials

Materials used were similar to those in Experiment 1, with the following exceptions. For both tasks, the target was a letter ‘E’ measuring 0.25° × 0.25° (RGB colour coordinates: 0, 0, 0) that was oriented upwards or downwards. The cue on the exogenous task was expressed by a 0.06° × 0.06° increment to the thickness of the outline of the left or right placeholder square, or both placeholder squares simultaneously for neutral cues.

#### Procedure

Participants completed both the endogenous and exogenous tasks, with the task order counterbalanced across participants. The endogenous cueing task was identical to the Experiment 1 task with the exception of the target type and associated judgement. Participants indicated the orientation of the letter target using the ‘up’ and ‘down’ keys (see Fig. 5).

In the exogenous cueing task, there were three conditions (invalid, neutral and valid) and four cue-target SOAs (100 ms, 200 ms, 500 ms and 800 ms). The addition of a smaller SOA (100 ms) was made based on results from a prior pilot study of the exogenous task, which suggested cueing benefits emerged at shorter SOAs – presumably due to the reflexive nature of exogenous cues that typically lead to faster attentional orienting (Klein et al., 2009). There were 20 trials at each SOA and cue condition combination, yielding a total of 240 trials. The exogenous task was divided into four equal blocks, with opportunities for breaks provided at the end of each block. Before commencing the task, participants were instructed to respond to the target letter as quickly and accurately as possible. Participants were also told that the cues would not always validly predict target location.

On each trial of the exogenous task, the participant was presented with a fixation cross flanked by an empty placeholder square on each side of the screen for 500 ms. This was followed by the presentation of a square with thickened borders on the left, right or both sides of the screen that acted as the three exogenous cues. The cue was presented for 50 ms, and the target letter ‘E’ was then presented 50 ms, 150 ms, 450 ms or 750 ms after the cue disappeared, depending on the SOA. The target remained on-screen until the participant indicated the orientation of the target using the ‘up’ or ‘down’ key. Once the participant responded, the target disappeared and was succeeded by an inter-trial interval of 500 ms, before the next trial was presented (see Fig. 5).

#### Design and analysis

As in Experiment 1, participants were selected for Low- and High-ALT groups on the basis of their overall AQ scores (Low-ALT group = bottom 20% of scores; High-ALT group = top 20% of scores). This yielded a total of 80 participants, with 42 participants in the Low-ALT group and 38 participants in the High-ALT group.

In both the endogenous and the exogenous tasks, data from participants whose target discrimination accuracy, cost score or facilitation score was more than 2.5 SDs below their respective overall group means were excluded from analyses. This resulted in the exclusion of ten participants (seven Low-ALT group, three High-ALT group) from further analyses.^2^ Descriptive statistics for the remaining participants can be seen in Table 2. IBM SPSS Statistics 26.0.0 was used to perform all statistical analyses.

**Table 2:** Descriptive statistics of Low- and High-ALT groups in Experiment 2

	Low ALT		High ALT	
*N*	35 (25 female, 10 male)		35 (23 female, 12 male)	
	Age, y	ALT score	Age, y	ALT score
Mean	20.4	89	21.0	134
*SD*	4.3	6.4	2.9	13.1
Range	18–35	68–95	18–33	123–180

### Results

Mean target accuracy was calculated as a function of ALT group (Low vs. High), cue-target SOA (200, 500, 800 ms) and Cue Condition (invalid, neutral, valid) and separately for the endogenous and exogenous tasks (see Appendix 2). In the endogenous cueing task, mean accuracy ranged from 95% to 98%, and an ALT group × SOA × Cue Condition mixed-design ANOVA revealed no significant main effects or interactions, all *F*s < .98, all *p*s > .31, all *η*^*2*^s < .004. In the exogenous cueing task, mean accuracy ranged from 95% to 98%, and an ALT group × SOA × Cue Condition mixed-design ANOVA also revealed no significant main effects or interactions, all *F*s < .87, all *p*s > .44, all *η*^*2*^s < .03. Thus, analyses of mean target accuracy for both cueing tasks suggest that interpretation of the RT data below is not compromised by speed-accuracy tradeoffs.

#### Endogenous cueing task

RT data for each Cue Condition and SOA combination were first screened for each participant, and individual RTs more than 2.5 SDs above or below their overall mean for each combination were excluded from further analyses. This accounted for 4.28% of trials in the Low-ALT group, and 4.89% of trials in the High-ALT group.

**Fig. 6:**
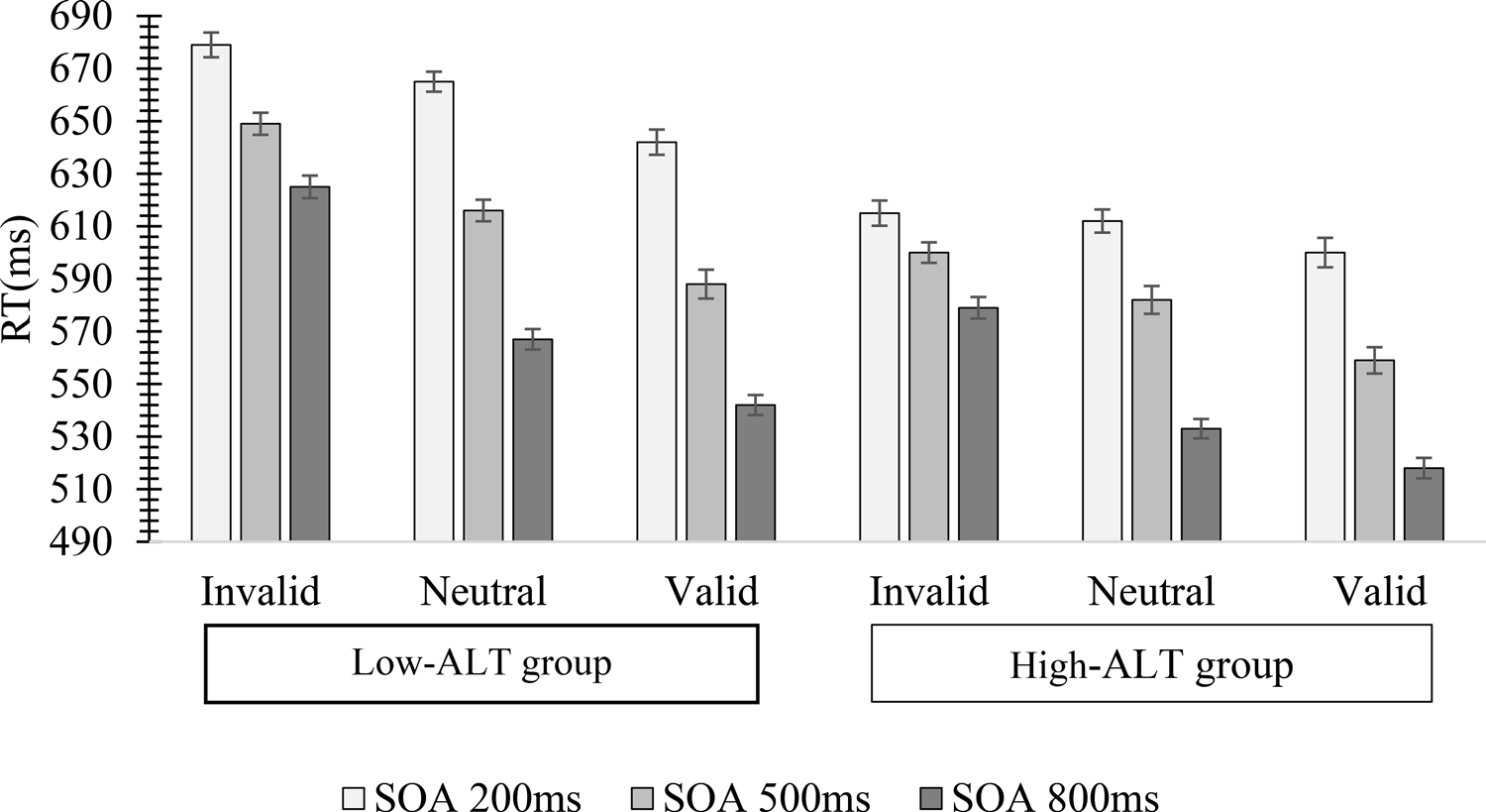
Mean reaction time (RT; ms) in the endogenous cueing task in Experiment 2 as a function of autism-like trait (ALT) Group, stimulus onset asynchrony (SOA), and Cue Condition. Values in parentheses represent standard errors of the mean

Outlier-removed means were then subjected to an ALT group (Low, High) × SOA (200 ms, 500 ms, 800 ms) × Cue Condition (invalid, neutral, valid) mixed-design ANOVA. This yielded significant main effects of SOA, *F*(2,148) = 235.08, *p* < .001, *η*^*2*^ = .76, Cue Condition, *F*(2,148) = 69.56, *p* < .001, *η*^*2*^ = .48, and ALT Group, *F*(1,68) = 7.53, *p* = .009, *η*^*2*^ = .10. The interaction between Cue Condition and ALT group was also significant, *F*(2,148) = 5.07, *p* = .008, *η*^*2*^ = .06. As evidenced in Fig. 6, post hoc t-tests revealed RTs in the High-ALT group were significantly shorter than those in the Low-ALT group for the invalid Cue Condition, *t*(68) = 2.51, *p* = .009, *d* = .36, the neutral Cue Condition, *t*(68) = 2*.*44, *p* = .007, *d* = .35, and the valid Cue Condition, *t*(68) = 2.33, *p* = .011, *d* = .34. No other interactions were significant, all *F*s < 1.35, all *p*s > .29, all *η*^*2*^s < .03.

We next examined conventional orienting scores by subtracting RTs on valid trials from those on invalid trials for each combination of ALT group and SOA. These orienting scores were then subjected to an ALT group × SOA mixed-design ANOVA, which revealed main effects of SOA, *F*(2,136) = 84.07, *p* < .001, *η*^*2*^ = .55, and ALT group , *F*(1,68) = 4.83, *p* = .03, *η*^*2*^ = .26, but no interaction, *F*(2,136) = .75, *p* = .55, *η*^*2*^ = .01. Notably, the orienting effect was smaller for the High-ALT group (*M* = 39 ms, *SE* = 4.1) than for the Low-ALT group (*M* = 44 ms, *SE* = 3.7).

**Fig. 7:**
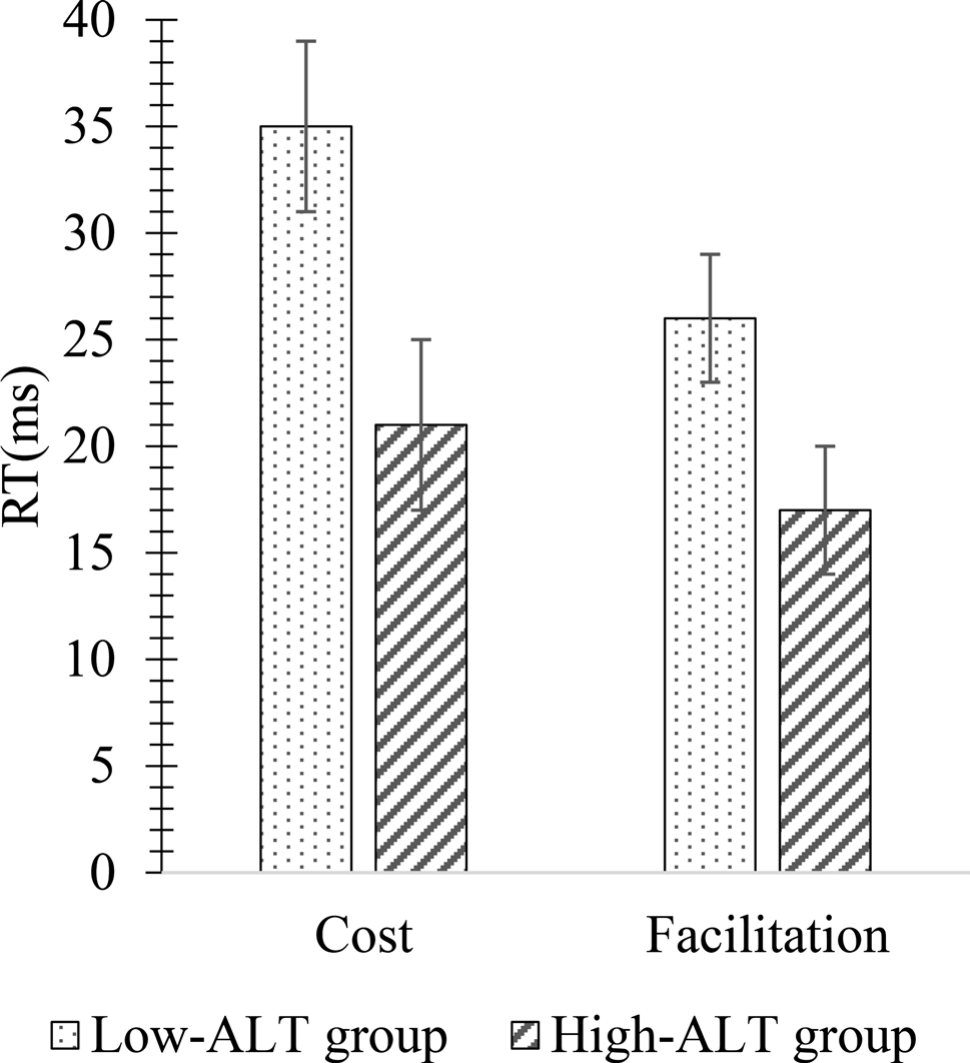
Cost and facilitation differences between High- and Low-ALT groups on the endogenous cueing task in Experiment 2 (collapsed across SOA). Error bars represent standard errors of the mean

Finally, cost and facilitation effects, which can be seen in Fig. 7, were calculated in a similar manner to that described in Experiment 1 and submitted to an ALT group × SOA × Cue Influence mixed-design ANOVA. There was a main effect of SOA, *F*(2,136) = 84.07, *p* < .001, *η*^*2*^ = .55. Bonferroni-corrected post hoc t-tests showed that the average of the facilitation and cost effects at the 200-ms SOA (*M* = 18 ms, *SE* = 2) was significantly reduced compared to the average effect at the 500 ms SOA (*M* = 45 ms, *SE* = 3), *t*(68) = 4.9, *p* < .001, *d* = .57, and 800 ms SOA (*M* = 44 ms, *SE* = 3), *t*(68) = 6.4, *p* < .001, *d* = .76. In contrast to the results of Experiment 1 where a significant interaction between ALT group and Cue Influence indicated a reduced cost effect but comparable facilitation effect for the High-ALT group relative to the Low-ALT group, in Experiment 2, a significant main effect of ALT group was observed, *F*(1,68) = 7.45, *p* = .008, *η*^*2*^ = .10, without qualification by an ALT group × Cue Influence interaction, *F*(2,136) = 1.65, *p* = .20, *η*^*2*^ = .02. Figure 7 shows how both the facilitation and effects were significantly smaller in the High-ALT group compared to the Low-ALT group.

#### Exogenous cueing task

RT data for each Cue Condition and SOA combination were first screened for each participant, and RTs more than 2.5 SDs above or below their overall mean for each combination were excluded from further analyses. This accounted for 4.15% of trials in the Low-ALT group and 4.58% of trials in the High-ALT group.

**Fig. 8:**
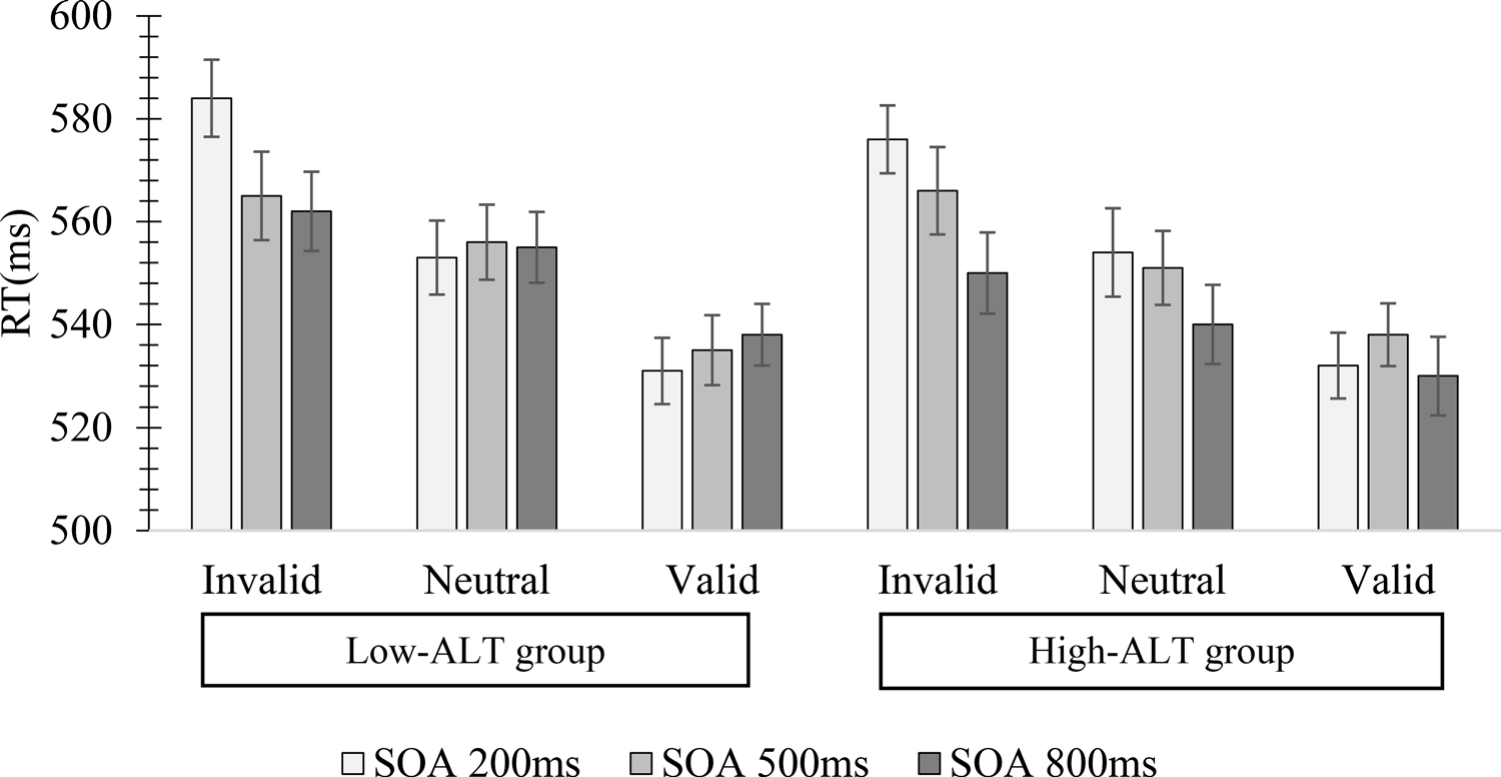
Mean reaction time (RT; ms) in the exogenous cueing task in Experiment 2 as a function of autism-like trait (ALT) group, stimulus onset asynchrony (SOA), and Cue Condition. Error bars represent standard errors of the mean

Outlier-removed means, which can be seen in Fig. 8, were then subjected to an ALT group × SOA × Cue Condition mixed-design ANOVA.^3^ This yielded significant main effects of SOA, *F*(2,136) = 122.49, *p* < .001, *η*^*2*^ = .64, and Cue Condition, *F*(2,136) = 12.07, *p* < .001, *η*^*2*^ = .16. There was no significant main effect for ALT group, *F*(1,68) = 2.68, *p* = .28, *η*^*2*^ = .04, and no significant interactions, all *F* < 1.72, all *p* > .43, all *η*^*2*^ < .006.

We next examined orienting by subtracting RTs on valid trials from those on invalid trials. These orienting scores were subjected to an ALT group × SOA mixed-design ANOVA. This analysis revealed a significant main effect of SOA, *F*(2,136) = 12.88, *p* < .001, *η*^*2*^ = .16. However, there was no significant main effect of ALT group, *F*(1,68) = .004, *p* = .46, *η*^*2*^ < .001, or ALT group × SOA interaction, *F*(2,136) = 1.67, *p* = .87, *η*^*2*^ = .02.

**Fig. 9:**
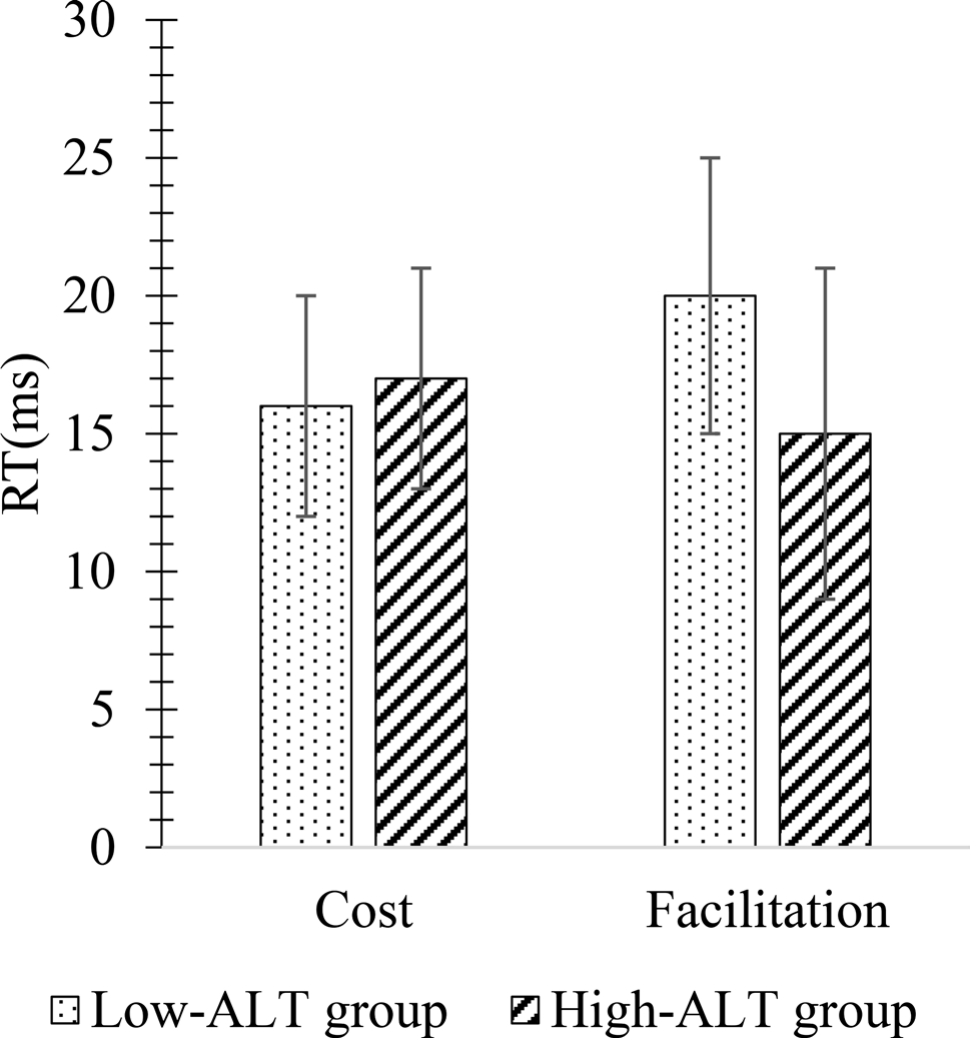
Cost and facilitation differences across High- and Low-ALT groups on the exogenous task in Experiment 2 (collapsed across stimulus onset asynchrony (SOA)). Error bars represent standard errors of the mean

Finally, cost and facilitation effects were calculated in the same manner as for the endogenous task (see Fig. 9). These scores were then submitted to an ALT group × SOA × Cue Influence mixed-design ANOVA. The main effects of ALT group and Cue Influence were non-significant, all *F*s < 2.19, all *p*s > .08, all *η*^*2*^s < .03, but there was a significant main effect of SOA, *F*(2,136) = 12.86, *p* < .001, *η*^*2*^ = .16. Post hoc Bonferroni-corrected t-tests showed that the average of the cost and facilitation effects was significantly greater at the 200-ms SOA (*M* = 28 ms, *SE* = 3) compared to the average effect at the 800-ms SOA (*M* = 14 ms, *SE* = 4), *t*(68) = 2.5, *p *= .011, *d* = .29. Also, the average of the cost and facilitation effects at the 500-ms SOA (*M* = 24 ms, *SE* = 3) was significantly greater than the average effect at the 800-ms SOA, *t*(68) = 2.9, *p* = .010, *d* = .35. No interactions were significant, all *F*s < .1.7, all *p*s > .26, all *η*^*2*^s < .02. Figure 9 shows cost and facilitation effects for the High- and Low-ALT groups on the exogenous cueing task.

Overall, the High-ALT group exhibited reduced cost and facilitation effects relative to the Low-ALT group on the endogenous task. The High-ALT group also had significantly shorter overall RTs on the endogenous task. No group differences in cost and facilitation effects were found on the exogenous task. Group differences in cost and facilitation effects also did not vary as a function of SOA on either task.

### Discussion

The objective of Experiment 2 was to compare endogenous and exogenous cueing with the same participants using an experimental design more akin to the designs of previous studies and that was expected to generate larger cueing effects. Our aims were to magnify the effects found in Experiment 1, and to compare performance between Low- and High-ALT individuals on the two forms of orienting. Findings from Experiment 2 further served to test the three theoretical positions used to account for differences in orienting: (1) difficulty in disengagement, (2) cue indifference, and (3) delay in orienting.

Findings from both the endogenous and the exogenous cueing tasks did not suggest a pattern of increased cost effect in individuals with High ALTs compared to those with Low ALTs as would be expected in the case of difficulty in disengagement. Results from both cueing tasks also did not demonstrate any differential impact of SOAs on cost and facilitation effects between the two ALT groups, inconsistent with a hypothesized delay in orienting.

However, the findings of reduced cost and facilitation effects in the endogenous cueing task for individuals with High ALT compared to those with Low ALT support the theory of cue indifference for individuals high on the autism continuum. On one hand, the reduced cost effect shown by the High-ALT group suggests that they were not misled by invalid cues as much as the Low-ALT group were, leading to a smaller cost effect as less time had to be spent re-directing attention to the target location. However, the trade-off for reduced cost appears to be reduced facilitation, as the High-ALT group also did not benefit from the valid cues as much as the Low-ALT group did, suggesting they also did not as readily orient to valid cues.

Contrary to the account of delayed orienting in autism, orienting differences between the High- and Low-ALT group did not vary across SOAs, unlike findings from previous studies that mostly compared autistic and non-autistic children on only exogenous cueing tasks (Flanagan et al., 2015; Townsend et al., 1996). In our experiment, participants were neurotypical individuals selected for differing levels of ALT. This stands in contrast to the sample used in most of the studies that found delays in orienting performance amongst autistic individuals or those with a genetic or biological predisposition towards autism (e.g., siblings of individuals with autism) compared to individuals without a family history of autism. Thus, it is possible that a delayed orienting effect varies across the autism continuum and may be more pronounced at the clinical end of the continuum.

Furthermore, the presence of differences between High- and Low-ALT groups for endogenous but not exogenous orienting is inconsistent with studies such as that of Iarocci and Burack (2004), which showed similar performance across the two forms of orienting for autistic and non-autistic individuals. Indeed, the majority of studies that found similar orienting performance between autistic and non-autistic individuals recruited adults or older children around the ages of 9–15 years (Kawakubo et al., 2004; Todd et al., 2009; Van der Geest et al., 2001). Thus, our results are particularly notable for showing an ALT group difference in endogenous orienting with adult participants.

The divergence of our results from other findings likely is due to our inclusion of a neutral cue condition that allowed us to isolate cue benefits and costs, thus making our analyses more sensitive to differences arising from variations in autistic traits (Kawakubo et al., 2004). Indeed, in Experiment 1, the conventional analysis failed to reveal any ALT group differences in orienting, despite an ALT group difference in cost effect that was shown following isolation of cost and facilitation outcomes. In Experiment 2, while the conventional analysis did indicate an ALT group difference in orienting, it could not be used to isolate whether the differences came from changes to costs, benefits or both. In other words, the method of separating cost and facilitation outcomes is not simply an alternative to the conventional analysis, but rather provides additional information about orienting.

## General discussion

Our two experiments were intended to compare how orienting performance differs between individuals separated on the autism continuum, and at the same time, attempted to reconcile disparities in findings of past literature by adopting a different approach to analysing orienting performance. Results pointing to differences in orienting behaviour between the study’s samples of Low- and High-ALT adults, at least in endogenous orienting, appear in line with previous studies that mostly observed orienting differences in samples of children. Thus, our findings suggest it is likely that there are parallels between orienting performance in children and adults for groups separated on the autism continuum.

Findings from our two experiments also support the cue indifference explanation for orienting differences for individuals with High ALTs relative to those with Low ALTs in endogenous but not exogenous orienting. This outcome has important implications for theories regarding autism. The existing literature presents plenty of discussion on the broader nature of the socio-cognitive difficulties in autism, namely, whether these difficulties arise from bottom-up, perceptual factors or stem from a more voluntary, top-down origin (Cook et al., 2012; Ursino et al., 2022). Results from the present study are consistent with the top-down account in the context of visual orienting, supplementing a growing body of evidence that visual difficulties in autism occur due to impaired top-down control mechanisms.

For instance, in a predictive cueing task, Greenaway and Plaisted (2005) found that, in contrast to non-autistic individuals, autistic individuals failed to show an orienting effect when onset cues that capture top-down attention by informing the location of the upcoming target were used. However, autistic participants showed an orienting effect similar to non-autistic participants when colour cues that capture attention in a bottom-up manner were used. Evidence of diminished top-down processes has been observed in autistic individuals compared to non-autistic individuals on tasks relating to perception of faces (Loth et al., 2010) as well as on change blindness tasks where the expectation of scene-object relatedness failed to influence change detection accuracy and RT for autistic individuals in contrast to its effect for non-autistic individuals (Loth et al., 2008). Furthermore, Maekawa et al. (2011) demonstrated reduced P300 potentials (a marker of top-down attention), but normal visual mismatch negativity, a marker of bottom-up attention, in autistic individuals compared to non-autistic individuals. The attenuation of top-down influences suggests that poor executive control of attention might be implicated in the development of autism.

The effect of cue indifference is one that has been documented in the literature, but mostly in the context of social cues. Haworth et al. (2016) recorded and analysed gaze and posture movements of autistic children as they viewed a series of dynamic visual stimuli, and found that these children displayed an overall insensitivity to the motion of the stimuli. It was posited that this indifference towards motion in general contributed to the indifference towards biological motion, which subsequently leads to the social disinterest towards the actions and motives of other people. Likewise, Moriuchi et al. (2017) demonstrated that autistic children were able to fixate on the eyes of a target when directly cued to do so but preferred to look at other facial regions when more subtle cues were presented, indicating that the lack of eye contact seen in most autistic individuals was perhaps due to an issue of indifference rather than aversion. The current study thus provides additional evidence for the theory of cue indifference, suggesting that this indifference might not be specific to only social cues.

While our study indicates strong support for cue indifference in High-ALT individuals that appears to be mediated by top-down processes, this explanation does not seem to hold for exogenous cueing as well. This may be explained most easily by differences in the role of top-down control in the different types of cueing. Whereas endogenous orienting relies more heavily on volitional processes (e.g., choosing to use the central cue to direct attention), exogenous cues elicit a reflexive, automatic orienting response. Thus, exogenous cues are at least less likely to be influenced by cognitive control processes (Dugué et al., 2020). This implies that additional research will be required to understand whether or how orienting differences between autistic or High-ALT individuals and non-autistic or Low-ALT individuals influence performance on tasks that elicit more reflexive attentional shifts.

Another notable aspect of our results is that the High-ALT groups consistently responded faster to targets than the Low-ALT groups on the endogenous task. Faster overall RTs in autistic or High-ALT individuals have been reported in a number of studies and tasks (Almeida et al., 2010; Ashwin et al., 2017; O'Riordan et al., 2001). For instance, Almeida et al. (2010) found shallower visual search slopes for High-ALT than Low-ALT participants, which they interpreted as evidence that High-ALT individuals were less susceptible to processing non-target visual items. They argued this pointed to group differences in cognitive processes (e.g., local processing style) or traits (e.g., cautiousness or impulsivity when responding to target) associated with autism or High ALTs. We suggest that our finding of both faster overall RTs and evidence if favour of cue indifference during endogenous orienting points more strongly to an explanation in terms of cognitive factors for both phenomena.

It should also be noted that the validity of our findings may be limited by a number of factors. First, our study used Low- and High-ALT groups (i.e., the latter group was not selected based on clinical diagnosis), rather than comparing autistic and non-autistic individuals as was done in the majority of past studies. The High-ALT group received scores of 123–180 on the AQ according to the 1–4 scoring method proposed by Austin (2005), which is equivalent to scores of 23–32 obtained using Baron-Cohen et al.’s (2001) binary scoring method. While the range of 23–32 is lower than the cut-off score of 32 proposed by Baron-Cohen et al. (2001) to distinguish between autistic and non-autistic populations, other studies have proposed lower threshold scores ranging from 25 to 29 that offer a better balance between achieving satisfactory false negative and false positive rates (Broadbent et al., 2013; Pisula et al., 2013; Woodbury-Smith et al., 2005). Additionally, the high scores received by the High-ALT group does not mean that individuals in this group would have met the diagnostic criteria for autism. It is possible that comparison between clinical and non-clinical samples may produce novel findings that reveal different facets of orienting behaviour in autism. In addition, the element of directionality in both cue and target may be confusing for some individuals. Participants were required to interpret the direction of cues (left or right), and also respond to the direction of the target (up or down) within a short period of time. While this paradigm has been popular in past studies using the Posner cueing task, it may lead to interference between cue and target processing that could influence orienting responses. One way to check on this possibility in future studies would be to have participants interpret different information from cues and target (e.g., inferring direction from cues while responding to the identity of target letters like E and F). Finally, head rests were not used in the experiments. As the tasks were administered to multiple participants at the same time, the use of head rests was unfeasible, and participants were instead instructed to fixate their gaze on the centre of the screen. As this was consistent throughout the experiments, it is not expected that findings were influenced in a systematic manner. However, it must still be acknowledged that lack of control of viewing distance may introduce noise to the data, which may potentially reduce the reliability of tested effects.

Extending the current findings, it would be worthwhile to further explore how top-down processes modulate the use of cue information in High-ALT individuals. One example would be to examine whether High- and Low-ALT individuals differentially attach significance to cues based on the cues’ predictability. For example, it could be that High-ALT individuals attach lower salience to conditions where there is a likelihood of invalid cues compared to Low-ALT individuals, which subsequently influences the decision-making process to be more indifferent to cues. To test this possibility, cue predictability could be varied for the endogenous task (e.g., having conditions where 50%, 70% and 90% of the cues correctly predict target location). Other factors related to characteristics commonly noted in the autism presentation, such as comfort or tolerance of uncertainty, may also be explored in conjunction with the varying conditions of cue predictability.

A second area that warrants further investigation would be the applicability of cue indifference in the interpretation of social information. Taken together, a general indifference towards cues in endogenous orienting underscores the main characteristic of social difficulties in autistic (Amso et al., 2014). Social information is often complex and meaningful, requiring additional time and effort to interpret before action can be taken. As such, it shares parallels with non-social endogenous cues where top-down resources must be employed in order for the information to be processed. An overall disregard towards such cues, particularly during early development, could potentially set the foundation for indifference and subsequently inability in interpreting social information (Keehn et al., 2013). Nevertheless, the nature of social cues is complex, with much contention on whether they capture attention endogenously or exogenously (Pruett et al., 2011). Some studies, such as Kylliäinen and Hietanen (2004), have utilised neutral-cue trials in a social cueing experiment on young children and found social cues to elicit a reflexive pattern of orienting that is more in line with exogenous orienting. However, other studies have suggested a more complicated array of processes behind social cueing in autistic individuals, where autistic individuals only utilised social cues when these cues correspond to or occur in conjunction with the appearance of salient information (Ristic et al., 2005). Consequently, it is possible for social cues to elicit a different pattern of orienting behaviour contrary to the reduced cost and facilitation pattern observed in this study. Direct comparisons of cost and facilitation effects for non-social cues like the arrows used here and social cues such as eye gaze would therefore be the next step to understanding social orienting difficulties reported in autistic individuals.

In summary, outcomes from both experiments in this study indicate the presence of differences between Low- and High-ALT groups in endogenous visual cueing tasks. These differences suggest the High-ALT group is showing cue indifference, although further investigation is needed to understand the processes leading to the orienting behaviour exhibited by High-ALT individuals.

### Electronic supplementary material

Below is the link to the electronic supplementary material.

Supplementary file1 (DOCX 27 KB)

## Appendix 1

For the endogenous task of Experiment 1, performance accuracy was measured using the proportion of trials answered correctly (as indicated by correct identification of target orientation) for each combination of SOA and cue condition. These accuracy data (see Table 3) were subjected to an ALT group × SOA × Cue Condition mixed-design ANOVA. There were no significant main effects of SOA, *F*(2,104) = .06, *p* > .05, *η*^*2*^ = .001, ALT group, *F*(1,52) = 1.06, *p* > .05, *η*^*2*^ = .02, or Cue Condition, *F*(2,104) = 1.59, *p* > .05, *η*^*2*^ = .03 (see Table 3). Interactions between ALT group and SOA, *F*(2,104) = .22, *p* > .05, *η*^*2*^ = .001 as well as ALT group and Cue Condition, *F*(2,104) = .19, *p* > .05, *η*^*2*^ = .001, were not significant. The interaction between ALT Group, SOA and Cue Condition was also non-significant, *F*(4,208) = .40, *p* > .05, *η*^*2*^ = .001.

**Table 3:** Descriptive statistics for accuracy data in Experiment 1. Values in parentheses represent standard errors of the mean

	Low ALTs			High ALTs		
	Invalid	Neutral	Valid	Invalid	Neutral	Valid
SOA 200 ms	.95	.98	.98	.96	.96	.97
	(.020)	(.012)	(.013)	(.019)	(.014)	(.014)
SOA 500 ms	.98	.95	.98	.97	.96	.95
	(.011)	(.012)	(.014)	(.012)	(.011)	(.012)
SOA 800 ms	.97	.97	.98	.96	.96	.96
	(.013)	(.014)	(.014)	(.013)	(.021)	(.011)

*ALT* autism-like trait, *SOA* stimulus onset asynchrony

## Appendix 2

### Endogenous cueing task, Experiment 2

Accuracy data (see Table 4 for descriptive statistics) for this task were subjected to an ALT group × SOA × Cue Condition mixed-design ANOVA. There were no significant main effects of SOA, *F*(2,136) = .50, *p* > .05, *η*^*2*^ = .001, ALT group, *F*(1,68) = .35, *p* > .05, *η*^*2*^ = .002, or Cue Condition, *F*(2,136) = .97, *p* > .05, *η*^*2*^ = .003. Interactions between ALT group and SOA, *F*(2,136) = .42, *p* > .05, *η*^*2*^ = .002, as well as ALT group and Cue Condition, *F*(2,136) = .28, *p* > .05, *η*^*2*^ = .002, were not significant. The interaction between ALT Group, SOA and Cue Condition was also non-significant, *F*(4,272) = .30, *p* > .05, *η*^*2*^ = .001.

**Table 4:** Descriptive statistics for the accuracy data for the endogenous cueing task in Experiment 2. Values in parentheses represent standard errors of the mean

	Low ALTs			High ALTs		
	Invalid	Neutral	Valid	Invalid	Neutral	Valid
SOA 200 ms	.96	.97	.97	.95	.96	.96
	(.012)	(.012)	(.011)	(.020)	(.021)	(.011)
SOA 500 ms	.97	.97	.96	.95	.95	.97
	(.011)	(.010)	(.012)	(.011)	(.022)	(.010)
SOA 800 ms	.97	.98	.97	.96	.97	.98
	(.012)	(.010)	(.010)	(.012)	(.010)	(.012)

*ALT* autism-like trait, *SOA* stimulus onset asynchrony

### Exogenous cueing task, Experiment 2

Accuracy data (see Table 5 for descriptive statistics) were subjected to an ALT group × SOA^4^ × Cue Condition mixed-design ANOVA. There were no significant main effects of SOA, *F*(2,136) = .64, *p* > .05, *η*^*2*^ = .01, ALT group, *F*(1,68) = .78, *p* > .05, *η*^*2*^ = .01, or Cue Condition, *F*(2,136) = .86, *p* > .05, *η*^*2*^ = .02. Interactions between ALT group and SOA, *F*(2,136) = .51, *p* > .05, *η*^*2*^ = .001, as well as ALT group and Cue Condition, *F*(2,136) = .79, *p* > .05, *η*^*2*^ = .002, were not significant. The interaction between ALT Group, SOA and Cue Condition was also non-significant, *F*(4,272) = .45, *p* > .05, *η*^*2*^ = .001.

**Table 5:** Descriptive statistics for the accuracy data for the exogenous cueing task in Experiment 2. Values in parentheses represent standard errors of the mean

	Low ALTs			High ALTs		
	Invalid	Neutral	Valid	Invalid	Neutral	Valid
SOA 200 ms	.97	.96	.97	.96	.95	.97
	(.012)	(.011)	(.011)	(.012)	(.019)	(.013)
SOA 500 ms	.96	.97	.97	.95	.98	.98
	(.011)	(.013)	(.012)	(.013)	(.011)	(.014)
SOA 800 ms	.97	.96	.98	.97	.97	.98
	(.014)	(.018)	(.012)	(.010)	(.010)	(.012)

*ALT* autism-like trait, *SOA* stimulus onset asynchrony

## Footnotes

1 At the request of a reviewer, an examination of data from participants who were excluded due to low cost or facilitation effects can be found in the Online Supplementary Material (OSM) Analyses.

2 An examination of data from participants who were excluded due to low cost or facilitation effects can be found in the OSM Analyses.

3 For ease of direct comparison, it was decided to analyse data from SOAs of 200 ms, 500 ms and 800 ms in keeping with the endogenous task. An identical analysis was performed using SOAs of 100 ms, 500 ms and 800 ms for the exogenous task due to findings from a previous pilot task showing greater exogenous facilitation and cost effects at the shorter SOA. This analysis yielded an identical pattern of results to the one reported here.
