# Supplementary material for: Visuospatial cueing differences as a function of autistic traits
Source: Atten Percept Psychophys. 2024 Apr 1;86(4):1342–59. doi: 10.3758/s13414-024-02871-0 (PMC11093807; doi:10.3758/s13414-024-02871-0)
Supplement: Supplementary file 1 — Supplementary file1 (DOCX 27 KB) [file 13414_2024_2871_MOESM1_ESM.docx]

**Supplementary Analyses**

The supplementary analyses examine RT data from participants who were excluded from Experiments 1 or 2 due to significantly reduced facilitation and cost effects. Given that most studies in this research area have suggested that any cueing difference established as a function of autistic traits might be small, it is important to ensure that individuals are reliably performing the tasks as expected. Thus, the rationale for implementing this exclusion criterion stemmed from the need to obtain a sample who reliably exhibit the critical phenomena of interest in order to probe the impact of autistic traits. Nevertheless, in the interest of transparency, analyses of cost and facilitation data from participants who were excluded are presented in this supplementary section.

**Experiment 1**

Of the 23 participants who were excluded from the main analyses, 12 (5 High-ALT group; 7 Low-ALT group) had performance accuracy that was 2.5 SDs below their respective overall group mean (76.9%). The other 11 participants (5 High-ALT group and 6 Low-ALT group) were excluded as their cost or facilitation effect was more than 2.5 SDs below their respective overall group mean. To examine whether these 11 participants performed similarly to those included in the main analyses, we looked at cost and facilitation effects as a function of ALT group using identical analyses to those in Experiment 1.

As shown in Figure S1, mean facilitation and cost effects in the High-ALT group were somewhat lower than those in the Low-ALT group. Quantitatively, this pattern of results is similar to that reported in Experiment 1, particularly for cost effects. The data were then submitted to an ALT group x SOA x Cue Influence (facilitation, cost) mixed-design ANOVA which showed no significant main effects or interactions (all *F*s < .98, all *p*s > .40, all *η^2^*s < .01), likely reflecting the small participant numbers. Overall, it seems there is little compelling evidence that these participants performed differently to those included in the main analysis of Experiment 1.

**Figure S1.** Cost and facilitation differences between High- and Low-ALT groups for the 11 participants initially excluded on the basis of cost and facilitation effects in Experiment 1 (collapsed across SOA). Error bars represent one standard error of the mean.

**Experiment 2**

Of the 10 participants who were excluded from the main analyses, 6 (5 High-ALT group; 1 Low-ALT group) had performance accuracy that was 2.5 SDs below their respective overall group mean (78.2%). The other 4 participants (2 High-ALT group and 2 Low-ALT group) were excluded as their cost or facilitation effect was more than 2.5 SDs below their respective overall group mean. Data from these participants is shown below, although we did not conduct inferential statistics due to the extremely small sample size.

Mean facilitation and cost effects in the endogenous cueing task for the High- and Low-ALT groups are presented in Figure S2. As can be seen in the figure, group differences for cost scores are analogous to those in Experiment 2, although the facilitation effects are essentially identical for the two groups. Of course, it is difficult to offer any meaningful interpretations from these data given the small sample size, but it seems unlikely these participants differ meaningfully from those included in the main analysis in Experiment 2.

**Figure S2.** Cost and facilitation differences between High- and Low-ALT groups for the four participants initially excluded on the basis of cost and facilitation effects in the endogenous cueing task of Experiment 2 (collapsed across SOA). Error bars represent one standard error of the mean.

Mean facilitation and cost effects in the exogenous cueing task for the High- and Low-ALT groups are presented in Figure S3. As with the endogenous task, the group difference for cost scores quantitatively mirrors the difference reported in the main analysis for Experiment 2, while the facilitation effect is larger in the High-ALT group. Given the small sample sizes, it seems unlikely that these participants differ meaningfully from those included in the analysis in Experiment 2.

**Figure S3.** Cost and facilitation differences between High- and Low-ALT groups for the four participants initially excluded on the basis of cost and facilitation effects in the exogenous cueing task of Experiment 2 (collapsed across SOA). Error bars represent one standard error of the mean.
